# Protecting against different subtypes of influenza viruses: a nanoparticle approach

**DOI:** 10.1038/s41392-020-0157-3

**Published:** 2020-04-24

**Authors:** Changhua Yi, Yongxiang Yi, Junwei Li, Pradeep Kumar Sacitharan

**Affiliations:** 1 Department of Infectious Diseases, The Second Hospital of Nanjing, The Affiliated Hospital of Nanjing University of Chinese Medicine, #1 Zhongfu Road, Nanjing, Jiangsu Province China; 2 Public Health and Therapy Center of Nanjing, 211113 Nanjing, China; 3 Department of General Surgery, The Second Hospital of Nanjing, The Affiliated Hospital of Nanjing University of Chinese Medicine, #1 Zhongfu Road, Nanjing, Jiangsu Province China; 4 College of Veterinary Medicine, Qingdao Agricultural University, 266109 Qingdao, China; 5 The Institute of Ageing and Chronic Disease, University of Liverpool, Liverpool, L7 8TX UK; 6 Department of Biological Sciences, Xi’an Jiaotong-Liverpool University, #111 Ren’ai Road, Suzhou Industrial Park, 215123 Suzhou, Jiangsu Province China

**Keywords:** Drug delivery, Infection

**A recent article by Wang et al. elegantly synthesized new nanoparticles consisting of cyclic guanosine monophosphate–adenosine monophosphate (cGAMP), which were administered alongside vaccines, to successfully deliver into the lungs of animals leading to protection against different subtypes of influenza infection without inflammatory side effects**.

**Fig. 1:**
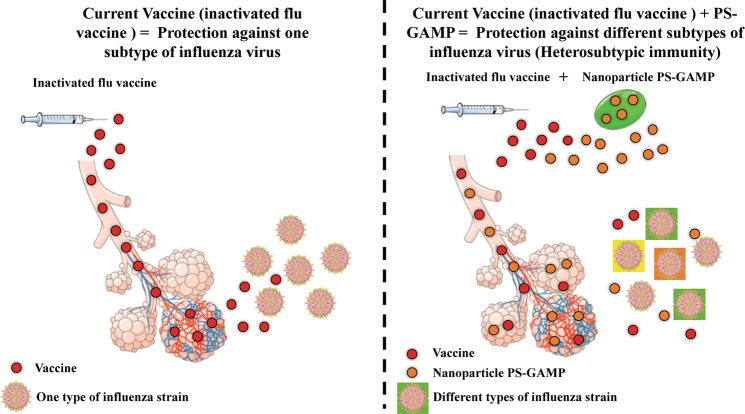
Current vaccines (inactivated flu vaccines) help by boosting immunity against one subtype of influenza virus. However, this approach means seasonal and regular vaccines are required to combat the constant mutations caused by antigenic drift. Wang et al. showed augmenting current vaccines with a new nanoparticle (PS-GAMP) that can activate long-term immunity against different subtypes of influenza viruses (heterosubtypic influenza immunity). The results also hold promise to use this type of technology for universal vaccines against other respiratory viruses

Infections are common among everyday life.^1^ However, certain infectious agents can cause widespread health and societal burdens.^2^ A common problem with current vaccine treatments is the inability to protect against the infection when it has mutated owing to antigenic drift.^3^ Hence, there has always been a long quest in the field of infection to discover treatment options that will produce cross-protection (heterosubtypic immunity) to infectious stereotypes. A method of providing this type of protection is to activate the immune system in the early phase of the infection without a chronic pathogenic inflammatory response to the foreign particles (Fig. 1).

Type I interferons (IFN-Is) are produced by alveolar epithelial cells (AECs) and immune cells to protect against infection.^4^ These genes are activated by STING (stimulator of interferon genes). Past attempts to target STING have failed owing to the inability to deliver STING agonists into the cytosol of AECs without breaching the integrity of the pulmonary surfactant (PS). Wang et al. hypothesized that nanoparticles consisting of cGAMP, an agonist of STING alongside an adjuvanted H1N1 vaccine, will induce an immune response by CD8^+^ T cells, which can yield heterosubtypic immunity.^5^

The authors at first successfully synthesized nanoparticles with the correct size, function, and safety. They tested the effects of the nanoparticles in vitro (bone marrow-derived macrophages) and in an in vivo model of infection. These results imply that the nanoparticles may be metabolically stable and have efficacy in humans, but more clinical trials are required. The authors also robustly showed that the protective effects of the nanoparticles did not occur in STING-deficient mice confirming that cGAMP, rather than any other constituents, was responsible for PS-GAMP adjuvanticity. The authors then went on to demonstrate that the nanoparticles uptake was specific to the lung, which suggested that there is a natural and molecule-specific mechanism of particle clearance in the lung that would sustain the integrity of the PS and alveolar epithelial barriers.

The next set of experiments demonstrated that the uptake of nanoparticles was only confined to the lungs of mice. In addition, there was no adverse inflammatory response in any body part and serum cytokines (IFN-β, IFN-γ, interleukin (IL)-6, IL-10, and tumor necrosis factor-α) levels did not change alongside mouse body weight and temperature. Moreover, the inclusion of PS-GAMP nanoparticles in the vaccination fully protected mice and ferrets from homologous viral challenges as early as 2 days after immunization in models of infection. Wang et al. went on to use gap junction blocking drugs to show that there is a gap junction–mediated flux of cGAMP to AECs from alveolar macrophages (AMs). These results empathised that the AECs rather than AMs appear to be essential in orchestrating innate and adaptive immune responses in the respiratory system during viral infection and determining the potency of PS-GAMP.

The final set of experiments in the article were conducted to determine whether the PS-GAMP nanoparticles alongside the influenza vaccination can protect against a number of different subtypes of the influenza viruses (heterosubtypic H3N2, H5N1, and H7N9 viruses). Mice receiving vaccines together with PS-GAMP were protected from challenges of different subtypes of the influenza viruses by CD8^+^ T_RM_ cells, rather than circulating memory CD8^+^ T cells. Most importantly, these in vivo results suggested that the nanoparticles plus vaccine approach was similarly effective for both influenza A and B viral vaccines. Finally, the authors successfully repeated the long-term heterosubtypic protection induced by the dual PS-GAMP and vaccine treatment in a ferret model of infection.

In summary, Wang et al. elegantly demonstrated nanoparticles consisting of cGAMP, an agonist of STING, helped augment adjuvanted H1N1 vaccine–induced humoral and CD8^+^ T cell immune responses. This early activation did not elicit a pathogenic inflammatory response but in fact protected against the H1N1 and heterosubtypic H3N2, H5N1, and H7N9 viruses for at least 6 months while maintaining lung-resident memory CD8^+^ T cells. The authors further showed that, when AECs lacked Sting or gap junctions were blocked, PS-GAMP–mediated adjuvanticity did not function in vivo. Overall, the studies by Wang et al. will be central to how new vaccines and augmented therapies will be designed and administered in the future. The ferret models used throughout the paper suggest the potential for the approach in humans, but clinical trials are required. Nonetheless, these fundamental key findings can possibly help create a new generation of treatments that can protect against viral mutations in early infection to stop the spread of different respiratory viruses, not only influenza viruses, and cease global pandemics.
